# Roles and Research Trends of Neuroscience on Major Information Systems Journal: A Bibliometric and Content Analysis

**DOI:** 10.3389/fnins.2022.872532

**Published:** 2022-08-03

**Authors:** Chien-Liang Lin, Zezhou Chen, Xinyue Jiang, Guan Lin Chen, Peiqi Jin

**Affiliations:** ^1^College of Science and Technology, Ningbo University, Ningbo, China; ^2^Research Center for Ningbo Bay Area Development, Ningbo University, Cixi City, China; ^3^School of Economics, Zhejiang University of Technology, Hangzhou, China; ^4^College of Computer Science and Technology, Zhejiang University of Technology, Hangzhou, China; ^5^Department of Distribution Management, Shu-Te University, Kaohsiung, Taiwan; ^6^School of Foreign Language, Zhejiang University of Technology, Hangzhou, China

**Keywords:** NeuroIS, neuroscience, bibliometric study, management information systems, VOSviewer

## Abstract

Over the past decade, neuroscience has been integrated into information systems as a new methodology and perspective to study and solve related problems. Therefore, NeuroIS has emerged as a new cutting-edge research field. This review aimed to identify, summarize, and classify existing NeuroIS publications through knowledge mapping and bibliometric analysis. To effectively understand the development trend of NeuroIS, this study referred to the journal selection index of the Association of Business Schools in 2021 and journals above three stars in the field of information management as the main selection basis. A total of 99 neuroscience papers and their citation data were included from 19 major information systems journals of SCI/SSCI. This study analyzed bibliometric data from 2010 to 2021 to identify the most productive countries, universities, authors, journals, and prolific publications in NeuroIS. To this end, VOSviewer was used to visualize mapping based on co-citation, bibliographic coupling, and co-occurrence. Keywords with strong citation bursts were also identified in this study. This signifies the evolution of this research field and may reveal potential research directions in the near future. In selecting research methods and analysis tools for NeuroIS, content analysis was used to further conclude and summarize the relevant trends. Moreover, a co-citation network analysis was conducted to help understand how the papers, journals, and authors in the field were connected and related, and to identify the seminal or pioneering major literature. For researchers, network maps visualized mainstream research and provided a structural understanding of NeuroIS. The review concludes by discussing potential research topics in this field.

## Introduction

The application of cognitive neuroscience in information management is a new research approach in the field of information management developed in recent years and is also known as the neural information system. Dimoka et al. (2012) defined a neural information system by combining theories, methods, and tools of cognitive neuroscience (such as EEG, fMRI, and ERP) to investigate problems in the field of information systems. Such a practice can overcome the shortcomings of conventional questionnaire surveys in explaining behavioral science and enable people to understand the factors influencing their deeper-layer behaviors through brain waves or EEG technology (vom Brocke and Liang, 2014). Since 2010, information systems have been discussed in many top journals on information systems, such as MIS Quarterly, to investigate neuroscience. For example, Dimoka (2010) investigated the online trust mechanism using neuroscientific methods, and Riedl et al. (2010) explored the differential influence of gender on trust, both of which were innovative studies in this field. Moravec et al. (2019) conducted behavioral experiments on social media users by means of EEG or brain waves to understand whether they could detect fake news on social media and whether the signs of fake news would affect their cognition and judgment. More accurate physiological data were acquired with cognitive neuroscientific methods to examine the behavioral variables in information management and e-commerce in the past, which was conducive to more accurate scientific findings and effectively compensated for the shortcomings of conventional research approaches in social behavioral science.

In the past years, information system researchers have mainly conducted questionnaire surveys to analyze the investigation and interview data for the final research results. However, affected by social expectations and subjective prejudice, respondents could not answer the questionnaires based on their true feelings (Liang and vom Brocke, 2014). Compared with the experimental design of neuroscience, some problems are still seen in the traditional experimental design, such as a certain degree of bias between the experimental environment and the real situation, which leads to reduced external validity. In addition, because the measurement objects are people whose behaviors vary greatly, it is difficult to have appropriate measurement tools, which can easily cause errors. However, NeuroIS is mainly used to complement existing IS research and neurophysiological tools to provide reliable data difficult or impossible to acquire from conventional ones (Dimoka et al., 2012). In recent years, NeuroIS has received increasing attention in the field of information management. For example, the Journal of the Association for Information Systems released a special issue titled “Methods, Tools, and Measurement in NeuroIS Research” in 2014, which contributed to the NeuroIS methodology. The Journal of Management Information Systems also released a special issue titled “Neuroscience in Information Systems Research” in the same year, defining the future focus in the discussion on rigorous NeuroIS research methods.

NeuroIS is an emerging discipline that has been attached with importance in the past 10 years, but beginners of NeuroIS often know nothing about where to start their research in this field. In reviewing previous studies on neuroscience, there have been studies on the application of neuroscience in management and entrepreneurship, although only coupling analysis and Co-Citation analysis were used (Cucino et al., 2022). Then, there have been articles explaining the development approach and core citation of neuroscience with applied bibliometrics and Bradford’s law released in the core journal, putting forward a specific statement for the overall approach of neuroscience (Yeung et al., 2017). Later, Kocak et al. (2019) discussed the trend analysis of research institutes and authors in Turkey in Scientometrics, a journal on bibliometrics, and the research approach and future research context by means of the knowledge graph. It can be learned that there have been a lot of literature discussions on neuroscience. As the application of information systems in neuroscience is a brand-new discipline, and relevant development has never been discussed in previous studies, this study investigates the current development of NeuroIS, and the methods applied in this field. Bibliometrics methods and VOSviewer software will be used in this study to analyze the research process and future development trends in this field. This study intends to solve the following problems: (1) To understand the current situation of NeuroIS, (2) to investigate the research approaches, experimental methods, research focuses, keyword evolution, keyword prominence, and other trends in NeuroIS in recent years, and (3) to investigate the co-citation of literature on NeuroIS. By reference to important journals, this study will give NeuroIS researchers an introduction to the current research development of NeuroIS and how to enter this field.

## Literature Review

Bibliometrics is the study of the external characteristics of bibliographic data and involves using mathematical and statistical methods to analyze the quantitative relation, distribution structure, and variation law of specific literature. Such analytical methods are often used to infer quantitative changes in a particular research discipline and are therefore useful for identifying publishing patterns on a certain topic and publishing trends within a discipline (De Bakker et al., 2005; Lee and Hew, 2018). Bibliometric analyses enable scholars to visualize data and observational results obtained from the literature, ensuring the high quality of the analysis and providing ample opportunities to utilize the information of all documents (De Rezende et al., 2018).

Many well-developed tools, such as VOSviewer, CiteSpace, SALSA, and PRISMA, are available for bibliometric analysis. Applicability and operability must be considered when selecting bibliometric analysis tools (Xu et al., 2021). Compared to SALSA and PRISMA, VOSviewer and CiteSpace are easier to operate and do not require programming skills; thus, these two tools are popular for rapid bibliometric analysis (Aria and Cuccurullo, 2018). VOSviewer can manage large amounts of data, has an excellent mapping function, and meets various research requirements (Donthu et al., 2020). CiteSpace can generate burst detection algorithms and time-zone views that change over time, supporting investigations into future research predictions and hotspot mutations (Kleinberg, 2003). These tools have been applied in a series of analyses, including those related to publishing and citation trends (Cunill et al., 2019).

In terms of bibliometric research, Su et al. (2020) applied Lotka’s law and Bradford’s law to explain the development status and application of social networks, analyzing the distribution of research productivity among researchers in the field of social network analysis. Zhang et al. (2020) discussed the research hotspots and development directions of the ecotourism economy, and Wu et al. (2021) applied bibliometrics to explore China’s COVID-19 pandemic policy patterns. Tang et al. (2021) systematically reviewed the publishing patterns of artificial intelligence and online learning research, focusing specifically on core periodicals, countries, discipline development, and cocitation network analysis. Galvez-Sanchez et al. (2021) analyzed research progress in the field of financial inclusion and conducted bibliometric analyses to identify scientific knowledge, trends, and future research directions with regard to financial inclusion. Furthermore, Mumu et al. (2022) applied bibliometric methods to analyze the development of the literature on e-commerce trust. Dolhey (2019) performed bibliometric analyses to discuss research trends related to entrepreneurial intention. These studies demonstrate how bibliometrics have been widely applied in various disciplines to address different issues (Miau and Yang, 2018; Kocak et al., 2019; Song et al., 2020; Gao et al., 2021). The current study focused on analyzing the Neuro-Information-Systems (NeuroIS) literature, with in-depth knowledge graph mining used to determine research hotspots and evolution trends.

## Data Collection

Since 2009, the NeuroIS Retreat Conference has been held every year to promote the development of NeuroIS (Riedl and Léger, 2016). At the International Conference on Information Systems, Dimoka et al. (2009) delivered a speech entitled “NeuroIS: Hype or hope?” The introduction of the conference paper explains in detail how NeuroIS should help scholars in the information systems field to conduct important studies. Riedl et al. (2010) further explained this definition of NeuroIS. Because NeuroIS has been valued and studied by the information systems field since 2009, the time period covered was limited from 2010 to 2021. Journals of SSCI and SCI included in the Web of Science (WOS) were the main literature data resources. The WOS offers high-quality literature and is frequently used in bibliometric research (Su et al., 2020; Jia et al., 2022). The search term rules in this study are as follows: TS = [(“fMRI” or functional magnetic resonance imaging) or (eye-tracking) or (event-related) or (electroencephalography or “EEG”) or (eye fixation related potential or “efrp”) or (neurois) or (Neuroscience) or (neuro information systems) or (neuro information systems)]. All keywords were connected by an OR. To ensure high quality of literature, the research scope of journals is limited to journals with more than three stars in the field of Information Management in the Academic Journal Guide, which was proposed by the Chartered Association of Business Schools (ABS) in 2021. As a result, 20 information systems journals were searched, and 101 articles were obtained.

**FIGURE 1:**
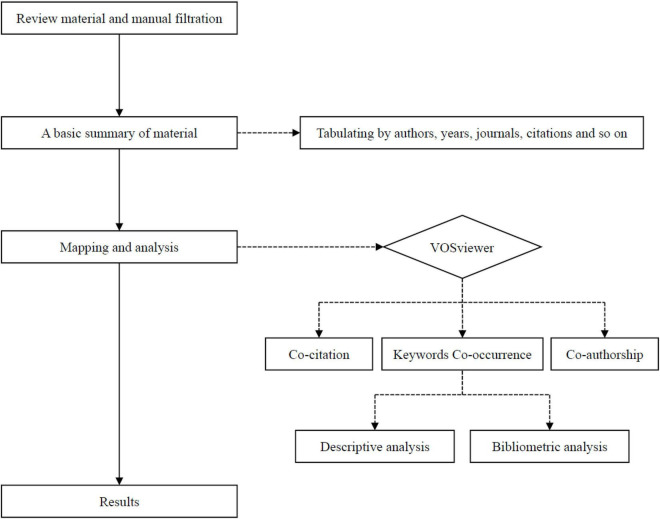
The article selection process for bibliometric mapping analysis.

Moreover, the type of literature is limited to “articles” only. To reduce errors in the keyword search, irrelevant papers were excluded. The deletion rule is based on Wang and Ngai (2020) and Jia et al. (2022). The method in which Taskin and Al (2019) conducted a standardized process of data cleaning, including the standardization process of author names, was used as a reference in this study. Since different journals present author names in different ways, errors may occur during data collection. Therefore, author names were uniformly verified, and improper author names were unified. Additionally, in some cases, publications without DOI may cause consistency in the calculation of cocitation analysis using the software. To ensure the consistency of the most cited publications in the dataset, several integrations were conducted to unify repetitive publications. Moreover, the consistency of keywords is also an important part of the data analysis. In particular, there are problems such as plural, verb change, or part-of-speech difference in some keywords, such as behavioral intention, behavior intention, and behavior intention. The step was to examine the abstract of each paper manually with the target subject through two-way confirmation. Irrelevant articles would be deleted. Finally, two papers that did not conform to the theme were removed, leaving a total of 99 articles. Subsequent data analysis was based on 99 papers. The process framework of this study is illustrated in Figure 1.

## Results

### Publication Trends

**FIGURE 2:**
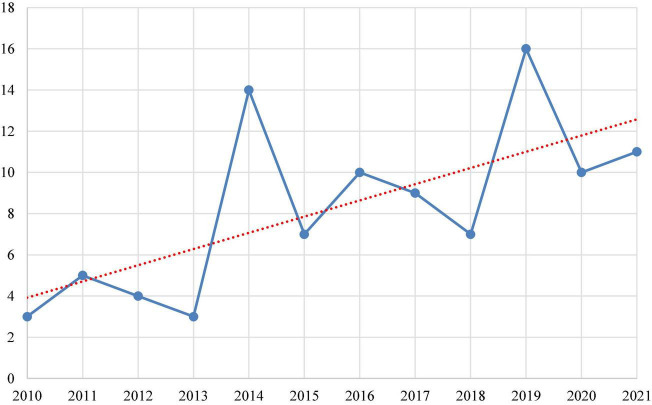
Number of publications per year from 2010 to 2021.

First, by comparing the number of published papers, the change in popularity of this topic can be revealed. Figure 2 shows the number of articles published annually from 2010 to 2021 in the field of NeuroIS. It can be found that from 2010 to 2013, the number of papers per year was less than six, indicating that NeuroIS was at the beginning of exploration and the stage of establishing the basic framework. In 2014, there was a great increase in the number of studies, reaching 14 years of age. This phenomenon was related to the launch of Brain Research through Advancing Innovative Neuroethologies and the Human Brain Project by the US and the EU, respectively, in 2013. More importantly, the Journal of the Association for Information Systems published a special issue in 2014 with the theme of “Methods, tools, and measurement in NeuroIS research,” which contributed to NeuroIS methodology. Professor Ting-Peng Liang, the late former president of the Information Systems Society, also published “Special Issue: Neuroscience in Information Systems Research” in the Journal of Management Information Systems in 2014. Its purpose is to discuss the key methods of NeuroIS research in the future (Liang and vom Brocke, 2014). The NeuroIS Society was established in Austria in 2018 to fund more scholars and research projects in this field. In 2019, the number of published papers peaked at 16. Among the 16, six papers were published in the Journal of the Association for Information Science and Technology. Overall, from 2010 to 2021, the number of published studies increased gradually. The intersection of neuroscience and information systems has been a hot topic and a research frontier in these two fields.

### Descriptive Analysis

Table 1 lists the number of published papers and citations in the selected journals. Among them, the Journal of the Association for Information Science and Technology (JASIST) has the most papers (20 papers), and most of them have been published after 2015. In the field of information science, this journal focuses on the production, discovery, recording, storage, representation, retrieval, presentation, manipulation, dissemination, use, and evaluation of information. Tools and technologies for processing were also included. The impact factor for this journal in 2020 was 2.687. After JASIST, the Journal of Management Information Systems published 19 papers, and the citations were 832, far ahead of those of other journals. Nine papers in this journal were published in the special issue “Neuroscience in Information Systems Research” in 2014. This journal is included in the FT50 as one of the top journals in the field of information systems. MIS Quarterly, another top-level journal of information systems, evaluated as a four-star journal by ABS and listed in UTD-24, has published 10 papers with 686 citations. It is ranked No. 1 in the rank of the average number of citations per article. The most interesting journal is Decision Support Systems, which ranks third in terms of the number of papers published in one journal. The number of published articles was the most stable among all the journals. Every year, it publishes papers on neuroscience. This means that this journal has the highest acceptance of NeuroIS studies, and researchers can consider this journal when attempting to publish.

**TABLE 1:** The top 10 productive journals from 2010 to 2021.

Ranking	Journals	Documents	TC	D| TC
1	Journal of the Association for Information Science and Technology	20	226	11.3
2	Journal of Management Information Systems	19	832	43.79
3	Decision Support Systems	17	323	19
4	Journal of the Association for Information Systems	11	280	25.45
5	MIS Quarterly	10	686	68.6
6	European Journal of Information Systems	5	50	10
7	Information Systems Research	5	172	34.4
8	Internet Research	4	35	8.75
9	Information Systems Frontiers	3	15	5
10	International Journal of Electronic Commerce	2	111	55.5
11	Journal of Computer-Mediated Communication	2	42	21
12	Information Systems Journal	1	3	3

*TC, total citations; D| TC, average number of citations per article.*

### Co-author Network

Researchers contribute the most to research achievements and actively find innovative research directions in the field. Table 2 displays the authors who have published more than three articles and their citation counts. According to the statistics in the table, the number of published articles differs among scholars. Seventeen scholars published more than three articles, accounting for 2.91% of the total. Seven articles were published by one author. Of course, when reviewing the rules of Lotka’s law, it is found that the number of authors producing n papers is approximately 1/n^2^ of those producing one paper. This is the inverse square law of scientific productivity: another rule in this law is that of all authors in a given field, 60% will have produced only one paper (Lotka, 1926; Su et al., 2020). However, in the present study, this proportion was 82.5%. Therefore, compared with the definition of Lotka’s law, the results of this study are consistent with the conclusion that many scholars have only one publication. The fact that few scholars have high yields in NeuroIS studies indicates that this is the first time that most scholars have entered the field of NeuroIS. In terms of citations, Angelika Dimoka has published four articles that have been cited 548 times, ranking first. Riedl and René (No. 2) published six papers with citations of 428. Kirwan and Brock have published the most (seven papers) among these authors.

**TABLE 2:** Authors with more than three papers and their citations.

Ranking	Name	Research subject	Documents	Citations
1	Kirwan, C. Brock	Memory, Learning, fMRI	7	212
2	Riedl, René	NeuroIS, Technostress, Digital Transformation, Human-Computer Interaction, Trust in Technology	6	428
3	Anderson, Bonnie Brinton	NeuroIS, Behavioral Information Security, Social Networks, Software Adoption, Women in Technology	6	211
4	Vance, Anthony	Security, Usable Security, NeuroIS, Behavioral Information, Security, Information Security	6	211
5	Jenkins, Jeffrey L.	Human-computer Interaction, Information Systems Security	6	169
6	Eargle, David	Behavioral Information Systems Security, Human-Computer Interaction, Neuroscience Applications to HCI and information Security	5	181
7	Dimoka, Angelika	Decision Neuroscience, Neuroeconomics, Neuromarketing	4	548
8	Davis, Fred D.	Technology Acceptance, NeuroIS	4	355
9	Vom Brocke, Jan	Organizational Design, Digital Strategy, Process Innovation, Enterprise Architecture, Business Engineering	3	182
10	Burgoon, Judee K.	Interpersonal and Non-verbal Communication, Deception, New communication technologies	3	163
11	De Guinea, Ana Ortiz	IT use and impacts, Data analytics, Research methods	3	122
12	Gedeon, Tom	Responsive AI, Neural/Deep Learning, Responsible AI, Multimodal Signal Processing, Affective Computing	3	87
13	Nunamaker, Jay F., Jr.	Systems Analysis and Design, Collaboration Technology, Deception Detection	3	76
14	Djamasbi, Soussan	Human Technology Interaction, UX Research, Decision Support Systems, NeuroIS, Eye Tracking	3	71
15	Twyman, Nathan W.	Information Systems, Deception Detection, Collaboration, Transformative Technologies, HCI	3	61
16	Gregor, Shirley	Information Systems, Decision Support, Philosophy of Technology, Adoption of Technology, Innovation	3	53
17	Riaz, Amir	Knowledge Production, Knowledge Utilization, Human-Computer Interaction, Neuro-IS, Neuro-Management, Social Entrepreneurship	3	53

**TABLE 3:** Citations of documents (>100).

Rank	Author (Year)	Title	Citations
1	Dimoka (2010)	What Does the Brain Tell Us About Trust and Distrust? Evidence from a Functional Neuroimaging Study	232
2	Riedl et al. (2010)	Are There Neural Gender Differences in Online Trust? An fMRI Study on the Perceived Trustworthiness of eBay Offers	185
3	Dimoka (2012)	On the Use of Neurophysiological Tools in IS Research: Developing a Research Agenda for NeuroIS	137
4	Turel and Qahri-Saremi (2016)	Problematic Use of Social Networking Sites: Antecedents and Consequence from a Dual-Systems Theory Perspective	135
5	Wang et al. (2014)	An eye-tracking study of website complexity from \ cognitive load perspective	123
6	Dimoka et al., 2011	Research Commentary—NeuroIS: The Potential of Cognitive Neuroscience for Information Systems Research	121
7	Nunamaker et al. (2011)	Embodied Conversational Agent-Based Kiosk for Automated Interviewing	114

*Source: Web of Science.*

**TABLE 4:** Countries (regions) with more than four documents and their citations.

Ranking	Name	Documents	Citations
1	United States	49	1,846
2	Germany	16	529
3	Mainland China	16	353
4	Australia	14	327
5	Austria	9	456
6	Canada	9	310
7	South Korea	6	268
8	Taiwan	4	129

*Source: Web of Science.*

**TABLE 5:** Institutions with more than four documents and their citations.

Ranking	Name	Documents	Citations
1	Brigham Young University	10	245
2	University of Pittsburgh	6	197
3	Karlsruhe Institute of Technology	6	96
4	Temple University	5	595
5	University of Linz	5	421
6	Australian National University	5	99
7	Zeppelin University	4	389
8	University of Arkansas	4	355
9	University of Arizona	4	190
10	Zhejiang University	4	135
11	HEC Montréal	4	129
12	University of Michigan	4	107
13	The University of Texas at Austin	4	67
14	Texas Tech University	4	55

*Source: Web of Science.*

By listing the research topics, it can be seen that the tools and methods used in NeuroIS research were the main focus. According to these results, high-yield authors contributed significantly to the NeuroIS study. Two interesting conclusions were drawn based on the authors’ productivity. First, among the top five high-yield authors, three are from Brigham Young University. This means that this university has greatly invested in the research of NeuroIS, especially in core research institutions, such as the Neurosecurity Lab for Neuroscience Studies.

Second, Professor Fred D. Davis, the original author of the technology acceptance model, also began NeuroIS studies in 2009 and published a series of papers on the research design, tools, and methods (Dimoka et al., 2011, 2012). He even co-founded the NeuroIS Society and held a NeuroIS Retreat every year to collect papers on NeuroIS and discuss relevant research in NeuroIS.

**FIGURE 3:**
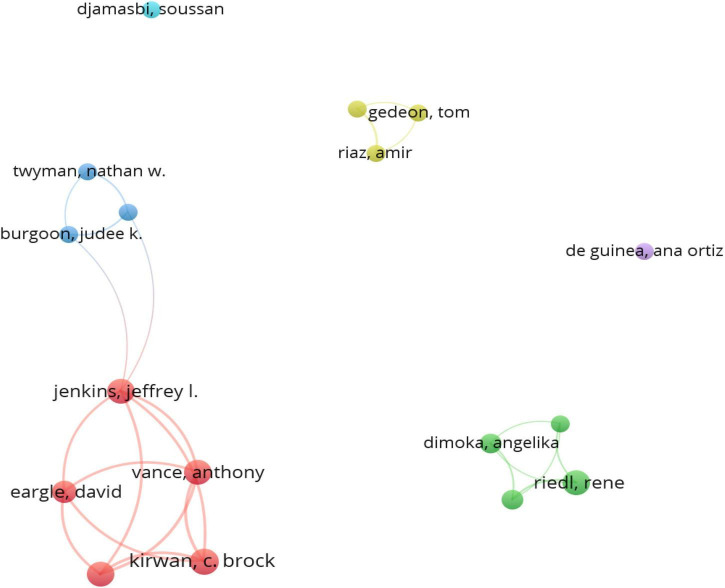
Co-author network of authors with more than three publications.

Figure 3 shows the corresponding author collaboration network. It can be seen obviously that these authors are grouped into six categories, and there is little cooperation among teams. The two teams were relatively conspicuous. The first was the team of Bonnie Brinton Anderson, Jeffrey L. Jenkins, Anthony Vance, C. Brock Kirwan, and David Eargle. Studies include those by Vance et al. (2014, 2018), Anderson et al. (2016a,b,c), and Jenkins et al. (2016). These studies were mainly applied to information security, risk profile, and other topics, which were analyzed using electroencephalography (EEG), fMRI, eye-tracking, and other tools. The second is the work of Angelika Dimoka, Ren é Riedl, Fred D. Davis, and others, who have published Dimoka et al. (2011, 2012) and Riedl et al. (2014). These studies discussed the definition of NeuroIS, tools, and methods and explored measurements of cognitive neuroscience.

In the analysis of citation rates, seven papers with high citation rates (more than 100 times) were screened through WOS. However, only the study of Turel (2016) is a highly cited paper. This study mainly investigates what drives the problematic use of social networks based on the dual-systems theory borrowed from cognitive neuroscience. This article contributes to the study of the dark side of IS use by conceptualizing problematic IS use and explaining its drivers and consequences. The results show that dual-systems theory is an appropriate theoretical perspective to explain problematic IS use, which is better than planned behavior-based models. In addition to the highly cited articles, the three most frequently cited articles are Dimoka (2010); Dimoka et al. (2012) and Riedl et al. (2010), with 232, 185, and 137 citations, respectively. Among them, Dimoka (2010) and Riedl et al. (2010) were both published in MIS Quarterly, the top journal of information systems. These two articles explored the problem of trust and were the earliest studies found in this study. Dimoka (2010) complemented psychometric measures of trust and distrust by observing the location, time, and level of brain activity. Trust and distrust activate different areas of the brain with different effects, explaining why they are different constructs associated with different neurological processes. In addition, using fMRI, Riedl et al. (2010) studied the biological factors in the difference between women and men in their decisions regarding trust. The results show that the brain areas that encode trustworthiness differ between women and men. The two articles both explored biological factors of human behavior in information systems research, contributing significantly to subsequent studies. The paper written by Dimoka et al. (2012) discussed the function of commonly used neurophysiological tools (e.g., EKG, eye-tracking, fMRI, and EEG) in information system research and their major strengths and weaknesses. It proposes some potential thematic areas and research topics: (1) development and use of systems, (2) IS strategy and business outcomes, and (3) group work and decision support. This article also provides some suggestions for the development of NeuroIS. Meanwhile, the study by Dimoka (2012), which was published simultaneously, focused on the application of fMRI in NeuroIS.

### Co-citation Network

**FIGURE 4:**
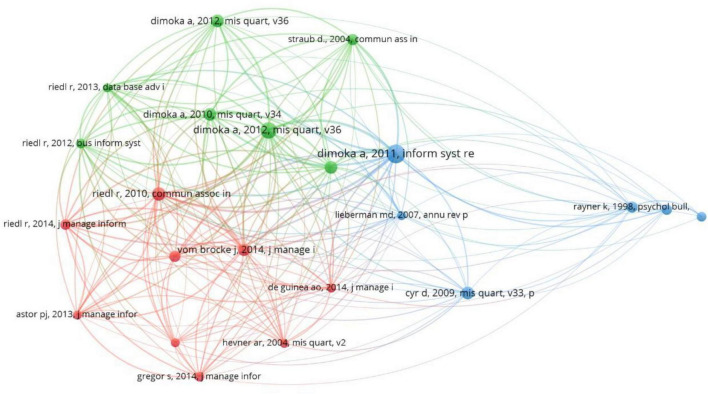
Co-citation Network (Citations ≥ 8).

A co-citation means that one paper is cited in two documents simultaneously. The co-citation network visualizes the relationship between cited articles and their source journals in the selected literature. Through the co-citation of articles, it is clear to see the key literature and pioneering literature referenced by different research methods on NeuroIS. The selected 99 articles cited 6,378 references from 2,644 journals. Figure 4 categorizes references into three groups. This study extracted articles that cited one of the three kinds of references to analyze their commonalities.

The first category (green) of citations is the literature review. For example, Straub et al. (2004) discussed the rigor of information systems research methods. In all the literature retrieved, there were 10 references of this kind, and most of them explored the potential research directions in NeuroIS at the overall level. vom Brocke et al. (2020) categorized future research of information systems into (1) IS design, (2) IS use, (3) emotion research, and (4) neuro-adaptive systems. This paper also presents possible challenges in these four fields and potential research directions in the future. Riedl et al. (2014) proposed that editors, reviewers, and authors should carefully consider reliability, validity, sensitivity, diagnosticity, objectivity, and intrusiveness.

The second category (red) focuses on research methodology. For example, Astor et al. (2013) introduced biosignals into information systems research, and Hevner et al. (2004) summarized design science in information systems. Eight articles cited this kind of literature, focusing on the introduction and improvement of research methodology. Hariharan et al. (2017) introduced the structure and function of the NeuroIS experimental platform Brownie according to the features of NeuroIS experiments. Leger et al. (2014) introduced the method of eye fixation-related potential (EFRP) to synchronize eye-tracking with electroencephalogram (EEG), and designed guidelines for researchers to use EFRP.

The keywords in the third category (blue) citations were eye-tracking or eye movements. Rayner (1998) reviewed research on eye-tracking in information processing, such as reading. Cyr et al. (2009) used eye-tracking to explore how Internet users regard human images as an element of website design in the context of e-commerce websites. Thirteen articles cited this type of literature, focusing on the mechanism of information processing by eye-tracking. For example, Hong et al. (2021) used eye-tracking to identify the role of animated banner ads in online marketing, whereas Jaeger and Eckhardt (2021) used eye movements to examine the safety awareness of phishing users. It is worth noting that many articles cited in these papers were set on the background of online marketing and searches (e.g., Huang and Kuo, 2011; Kim et al., 2015, 2016; Cheung et al., 2017; Xu and Zhang, 2019; Pfeiffer et al., 2020; Hong et al., 2021).

### Country (Region) and Institution Cooperation Network

According to the co-country network, this study analyzed data from 2010 to 2021. A total of 171 research institutions and 24 countries have conducted relevant research on NeuroIS, and eight countries (regions) have published at least four articles. Most articles were written by US researchers. American scholars participated in 49.49% (49 articles) of the publications cited 1,846 times. Mainland China and Germany ranked second in this field, accounting for 16.16% (16 articles), with 529 and 353 citations, respectively. Fourth, Australia has a number of published articles, accounting for 11.57% (14 articles), with 327 citations. Other countries are Austria (9), Canada (9), South Korea (6), and Taiwan (4). According to the comparison of the co-country network, the number of documents published and cited by the United States exceeds the sum of those of the second to fourth countries, proving the leading position of the United States in NeuroIS.

From the perspective of scholars, it can be seen that the countries of highly cited research institutions coincide with the countries of the most cited authors. For example, Angelika Dimoka is from Temple University (United States), Ren é Riedl is from the University of Applied Sciences Upper Austria and the University of Linz (Austria), and Bonnie Brinton Anderson, Jeffrey L. Jenkins, Anthony Vance, and C. Brock Kirwan are from Brigham Young University (United States).

### Analysis of Methodological and Neuroscience Tools

**FIGURE 5:**
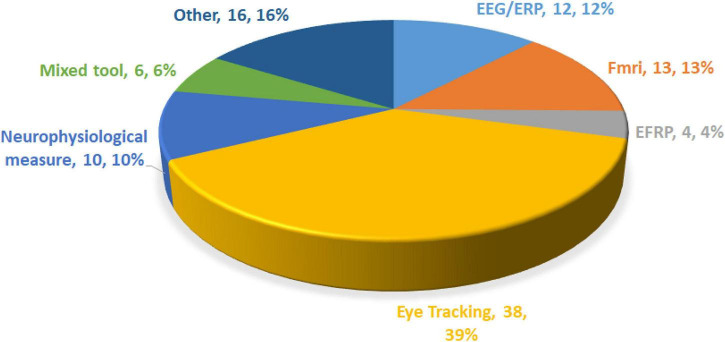
Analysis of neuroscience tools from 2010 to 2021.

In the application of the neuroscience method to information systems, the use of tools is an important reference basis and criterion for NeuroIS researchers to select reference research tools. Bibliometric analysis cannot explore the use of tools in neuroscience. As an improvement, a literature review was conducted in this study through the content analysis method. Bibliometric and content analyses were adopted by referring to the approach of Nagariya et al. (2021) in their research on the sharing economy in the hotel and tourism industry. The adoption of multiple analytical methods aimed to help reduce the bias associated with traditional literature reviews and expert interviews by complementing each other in a holistic, objective, and responsible manner. On the content analysis, this study was conducted in a case-by-case literature review. Two university professors and three research members were divided into two groups to read documents. First, three researchers conducted preliminary literature induction and classification, followed by a review by two university professors to ensure that the classification was accurate. Figure 5 shows the classification results for the research tools. This study found that neuroscience tools used in information systems mainly include eye-tracking, fMRI, EEG/ERPs, neurophysiological measures, EFRP, and a combination of the above tools.

Among the articles retrieved and shown in Figure 5, eye-tracking was the most frequently used tool, used in 38 papers (38.4%). In these studies, several main topics were discussed. For example, using eye-tracking, Djamasbi et al. (2011) found that baby boomers gazed more than Generation Yers on web browsing and online viewing, and their range of gaze was wider. Therefore, if the page did not present key information using a limited number of clear focuses at the top of the fold, Generation Yers were more likely to miss the key information than baby boomers. In subsequent studies, Wang et al. (2014) found that when users performed simple tasks, the fixation count and task completion time were at the highest level on highly complex websites. However, the fixation duration did not show a significant difference among websites with different complexities. This study explains this conclusion from a cognitive-load perspective. In addition, Willems et al. (2019) also used eye-tracking to analyze the effectiveness of online platforms using star ratings to show reputation and product or service quality. They found that reputation star ratings provided more supplementary information than alternative information. Moreover, users’ attention to different stars was asymmetric, which means that they paid more attention to situations with fewer stars. Reviewing previous research in the NeuroIS field, it was found that research on eye-tracking focused on online marketing and web search tasks.

The second most commonly used tool was fMRI, used in 13 studies (13.1%). Among these articles, Riedl et al. (2014) used fMRI to explore the differences and similarities between humans and avatars and between humans and humans based on multi-round trust games. They found that humans were better able to judge the credibility of humans and had less medial frontal cortex when judging avatar credibility. This brain region is crucial for predicting other people’s thoughts and intentions, and the trustworthiness learning rate is similar in both human and avatar interactions. It was found that when the product sold signaled a high reputation, consumers paid a higher premium for the product. Using fMRI, it was found that the seller’s high reputation signal triggered significantly stronger neural activity in brain regions associated with emotions in the prefrontal cortex. In these studies, fMRI analysis has mainly focused on exploring the neural basis of human information processing and decision-making.

The third was EEG/ERPs used in 12.1% of the papers. Specifically, Kuan et al. (2014) examined the role of two types of inducement information on group-buying sites: the number of people who have purchased a product and Facebook friends who like the product. It was found that both positive and negative information about the number of people who purchased had an asymmetrical effect on purchase intention. EEG observations revealed that information about the number of people who purchased was more associated with negative emotions, whereas information about Facebook friends’ likes was associated with positive emotions. Moravec et al. (2019) used the behaviors and EEG data of social media users in their study. They found most users could not distinguish between real and fake news and were more likely to believe news consistent with their political views, even if they were flagged as fake news, while titles that challenged their views rarely elicited cognitive activity (ignored). Only 17% of the users could detect fake news. Li et al. (2014) observed user attention and immersion in games using EEG data. Specifically, cortical activity on the left side of the DLPFC is associated with participation in games. Most studies using EEGs/ERPs have focused on the influence of emotion on decision-making.

Fourth is a neurophysiological measure. In addition to traditional neuroscientific methods, some researchers have adopted neurophysiological measures (10 studies). For example, Astor et al. (2013) designed a tool that can continuously display users’ emotional states through biofeedback, and adjust the difficulty of the decision-making environment according to users’ emotional states when they participate in decision-making games. EFRP was also used in the other four studies. Leger et al. (2014) introduced this method in their research on Information Systems. Adopting this method, researchers can use EEG and eye-tracking simultaneously to accurately capture users’ neural activity at the exact time they start to cognitively process a stimulus. In this paper, an example was given to show that EFRP could detect the attention response when the email notification popped up, the cognition and processing of the pop-up notification, and the motor planning activity designed to decide whether to open the email.

Finally, it is worth noting that six papers in the NeuroIS field adopted mixed tools. For example, Twyman et al. (2014a) built autonomous scientifically controlled screening systems (ASCSS). This system is used to detect whether individuals intentionally withhold information during a criminal investigation. Minas et al. (2014) used EEG, electrodermal activity, and facial electromyography to explore how team members process information received from text-based collaboration during group decision making. This provides neurological evidence for the underlying process of confirmation bias in information processing during online team discussions. These studies mainly focused on mixed research and effectively explored changes in consumer decision-making behaviors through measurements using multiple tools.

**FIGURE 6:**
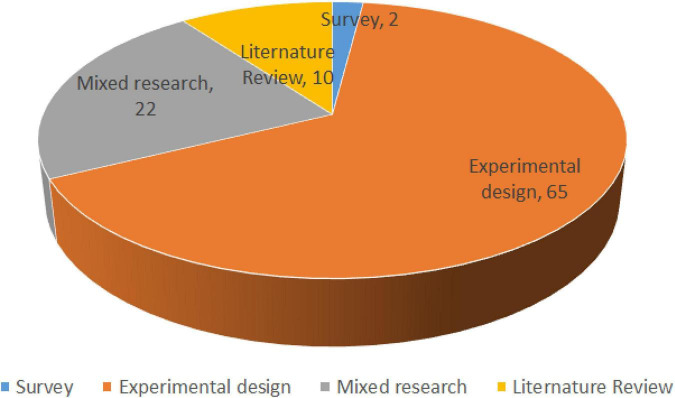
Analysis of research method from 2010 to 2021.

Furthermore, based on the previously excavated relevant literature (99 papers in total), the research methods were classified and summarized in Figure 6. With regard to research methods, the majority of papers (65 papers) obtained in this study used an experimental design, just two studies used surveys (Moody and Galletta, 2015; Turel and Qahri-Saremi, 2016), and 22 papers applied mixed research. In the mixed study, Schultheiss and Lewandowski (2021) adopted a mixed-research method, including pre-study interviews, an eye-tracking experiment, and a post-experiment questionnaire, to explore the effects of knowledge level and device screen size on users’ ability to distinguish between search results and advertisements when using search engines. Zhang and Zhang (2021) first used eye-tracking to obtain the time spent on reading words and subsequently used a neural network model to integrate the time spent on reading words into keyphrase extraction models. Ellison et al. (2020) adopted eye-tracking, survey, and interview methods to confirm that there was no difference in viewing duration between clicking in and not clicking on Facebook content. It can be seen that papers based on mixed research are relatively new, which means that mixed research may be a new trend in the NeuroIS field. In addition, 10 literature review-based studies were conducted for this study. Specifically, Riedl et al. (2014), Tams et al. (2014), and vom Brocke and Liang (2014) published papers on the special issues of JAIS and JMIS in 2014. The remaining literature reviews were distributed evenly between 2010 and 2021. These literature reviews are highly cited and influential and, to some extent, represent the trend that NeuroIS has begun to pay attention to the development and future integration of such research methods in this field in recent years.

### Co-occurrence Analysis: Keywords Network

**FIGURE 7:**
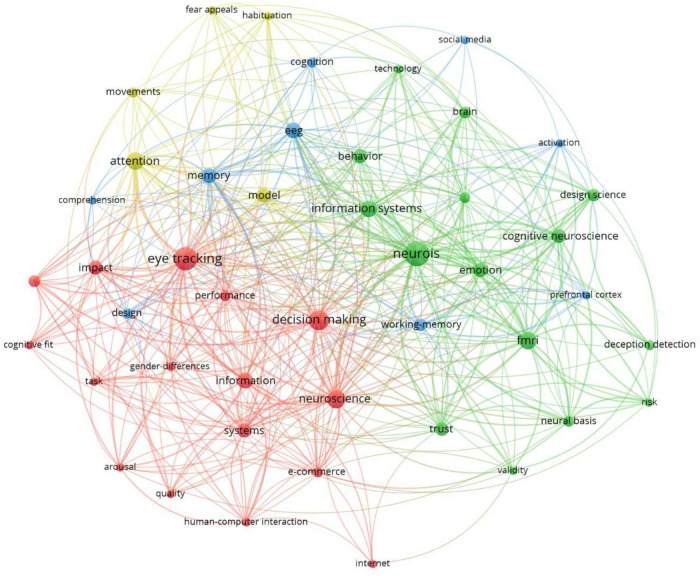
The Top 23 keywords with the strongest citation bursts from 2010 to 2021.

Keywords are intuitive indicators of core research content and topics in a specific scientific field. Research hotspots within a scientific field can be identified through keyword co-occurrence network analysis. A keyword co-occurrence network graph for the 99 articles analyzed in this study, generated using VOSviewer, is illustrated in Figure 7; keywords with a frequency greater than or equal to four were visualized. The size of the nodes is proportional to the number of keyword occurrences, with large nodes reflecting research hotspots in a certain field. The thickness of the lines linking nodes represents the strength of the correlation or co-occurrence between those keywords; that is, the thicker the line, the more frequently two keywords appear in the same literature. Different colors represent different clusters (i.e., research topics). Table 6 lists high-frequency keywords (i.e., those with a frequency greater than or equal to four).

**TABLE 6:** Cluster terms.

Clusters	Keywords
1	Activation (28), Arousal (20), Attention (18), Behavior (13), Brain (11), Cognition (10), Cognitive fit (7), Cognitive neuroscience (6), Comprehension (5), Deception detection (4), Decision making (4), Design (4), Design science (4), e-commerce (4), EEG (4), Emotion (4)
2	Eye tracking (32), Fear appeals (16), fMRI (15), Gender-differences (11), Habituation (11), Human-computer interaction (10), Impact (9), Information (8), Information search (7), Information systems (6), Internet (6), Memory (5), Model (4), Movements (4), Neural basis (4)
3	NeuroIS (13), Neuroscience (12), Performance (8), Prefrontal cortex (5), Quality (5), Research Agenda (4), Risk (4), Social media (4), Systems (4)
4	Task (16), Technology (11), Trust (5), Validity (4), Working-memory (4)

Cluster 1 reflected hotspot keywords in the neuroinformatic field, namely activation, arousal, attention, behavior, brain, cognition, cognitive fit, cognitive neuroscience, comprehension, deception detection, decision-making, design, design science, e-commerce, EEG, and emotion. According to these hotspot keywords, relevant research in neuroscience has focused on cognition-induced and conciseness-induced behavioral patterns. Brain, behavior, cognitive fit, and other cognitive neuroscience concepts have served as the foundation for cognitive comprehension, deception detection, and decision-making experiments. For example, Proudfoot et al. (2016) researched how variations in oculometric behaviors evolve during interactions with a deception detection system. Anderson et al. (2016a) investigated how neurobiology affects users’ habitual substantive understanding of security warnings and behaviors. Moreover, decision-making has always been a research hotspot. Twenty-seven of the 99 articles (27.84%) discussed in this study were centered on decision-making. For example, Xu et al. (2020) studied seller reputation by exploring emotions and power in online decision-making. Minas et al. (2014) investigated how team members process information received through text-based collaboration during a team decision-making process, as well as how information influences an individual’s preference for prediscussion. Day (2010) explored the effectiveness of applying the Needleman–Wunsch algorithm in identifying decision-making strategies using eye movement data. Moshfeghi and Pollick (2019) analyzed consumer demand for information retrieval and discovered brain regions related to inform decision-making through brain activity-related data. This study aims to understand consumers’ demand for information. Browne and Walden (2021) explored how consumers make decisions about information search and stop when making shopping behavior decisions and discovered brain activation through experiments to reveal the distributed regional network covered by the brain. These are also decision-making regions in which consumers do not participate in the search. Browne and Walden (2021) revealed the influence of excessive information consumption on a programmer’s information system design. The aforementioned research has focused on the effective use of cognitive neuroscience tools to promote behavioral pattern studies, thereby advancing the comprehensive development of various disciplines.

Cluster 2 reflected the hotspot keywords related to the research tools and basic dimensions of NeuroIS. These keywords were eye-tracking, fear appeal, functional magnetic resonance imaging (fMRI), gender differences, habituation, human-computer interaction, impact, information, information search, information systems, Internet, memory, model, movements, and neural basis. The aforementioned hotspot keywords covered research techniques, such as eye-tracking, movement, and fMRI, in addition to multiple basic research dimensions, such as fear appeal, human-computer interaction, gender differences, and habituation models (Anderson et al., 2016c; Vance et al., 2018; Bera and Poels, 2019; Kalgotra et al., 2019; Hong et al., 2021). Furthermore, much attention has been paid to information capture and comprehensive understanding in terms of research on information systems, information searches, and internalized memory. From the results of the cluster classification, the main research directions were as follows: Anderson et al. (2016b) used eye-movement tracking to learn how habituation occurs when people view security messages repeatedly, thus enabling us to design more effective security messages and reduce the security risks caused by excessive security warnings and reduced stimulus effects. Using fMRI, Warkentin et al. (2016) analyzed cognitive and emotional responses to fear appeals. They compared the arguments of fear appeal theory with the current neurological experience of internal personnel in the face of information security fear appeals. Through analysis, the effect of information system security can be better understood. Jaeger and Eckhardt (2021) used eye-tracking and surveys to analyze the security awareness of phishing emails. According to research findings, previous experiences could effectively improve the awareness of security warnings, but an effective content design could reduce the awareness of security. The results also verify that situational information can improve the awareness of threats and efficiency and the actual behavior response to phishing attacks. In these studies, basic tools and research dimensions in NeuroIS texts were comprehensively explored to broaden our understanding of human cognition, emotion, and behavior and capitalize on the potential of neuroscience knowledge and tools to further expand information systems research.

Cluster 3 reflected the hotspot keywords related to the microscopic inspection of related studies in NeuroIS research, namely NeuroIS, neuroscience, performance, prefrontal cortex, quality, research agenda, risk, social media, and systems. The high-frequency keywords of NeuroIS, neuroscience, and the research agenda, all reflected a microscopic focus on the control of the overall research and development direction of the NeuroIS field. Accordingly, when reviewing the research process, researchers must consider the future outlook, draft a blueprint for future development directions, and analyze the development potential of each research direction. Such studies have proposed views on the future NeuroIS research agenda and investigated its potentially considerable societal contributions (vom Brocke et al., 2020).

Cluster 4 reflected the hotspot keywords related to research techniques and methods and comprised task, technology, trust, validity, and working memory. Task, technology, trust, validity, and other abstract concepts represent current research directions and influences. According to the analysis of Cluster 4, these topics focused on the perspective of technology acceptance in several directions. By analyzing the technology acceptance model commonly discussed in behavioral beliefs, de Guinea et al. (2014) verified the potential implicit determinants of cognitive beliefs in traditional research through neuroscientific experiments. Their study made several contributions to the emerging field of receptive research and NeuroIS, including demonstrating the importance of emotional perception in mediating the effects of neurophysiological states on behavioral beliefs. On the other hand, some studies have used statistical methods and consumer neuroscience theories to take the perspective of technology and trust. For example, Karampournioti and Wiedmann (2021) used Parallax technology to determine how stories affect the user experience in online stores and variables related to branding and behavior. By applying parallax scrolling storytelling techniques, the online store improved the user experience of visitors at both the explicit and implicit information processing levels and enhanced the overall perceived appeal of the online store. Storytelling with parallax motion can effectively convey brand-related associations to consumers, enhance their explicit and implicit brand attitudes, and increase their willingness to pay higher prices. Therefore, according to the results of Cluster 4, this classification mainly focuses on the perspective of technology and trust, and relevant studies are also conducted from the perspective of technology adoption, purchase behaviors, and consumer intentions (Cheung et al., 2017; Mikalef et al., 2021), and then analyzed through the tools and methods of neuroscience.

### Keyword Evolution Analysis

To further elucidate research changes in the field, keywords with strong citation bursts were first identified (see Table 7).

**TABLE 7:** Keywords burst.

Keywords	Strength	Begin	End	2010–2021
Cognitive neuroscience	1.33	2010	2012	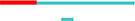
Information search	1.06	2010	2011	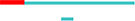
Price premium	0.98	2010	2013	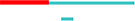
Neural basis	0.98	2010	2013	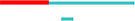
Emotion	2.12	2011	2014	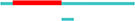
Impact	1.42	2011	2014	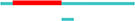
Deception detection	1.2	2011	2014	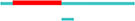
Performance	1.54	2013	2014	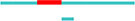
FMRI	2.76	2014	2016	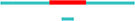
Working memory	1.91	2014	2016	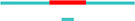
Acceptance	0.75	2014	2015	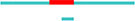
Internet	1.29	2015	2017	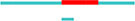
Decision support	1.04	2015	2016	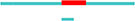
Behavior	1.55	2016	2017	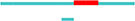
Information system	0.8	2016	2018	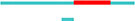
Design	0.73	2016	2018	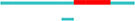
Eye tracking	1.39	2017	2018	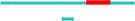
Criteria	0.77	2017	2019	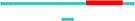
Memory	2.53	2018	2019	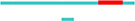
Neuroscience	2.25	2018	2019	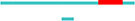
Cognitive fit	0.74	2018	2019	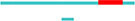
Consumer	0.62	2018	2021	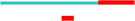
Comprehension	0.52	2018	2019	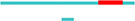

During the time course of keywords with strong citation bursts from 2010 to 2013, NeuroIS research articles were rare, and the general direction of research revolved around the field of cognitive neuroscience. Research directions mainly concentrated on information search (Huang and Kuo, 2011) and price premium (Dimoka, 2010; Ye et al., 2013). Although price premiums were no longer a focus after this citation burst period, information systems remain the primary topic of research.

The first research climax occurred in 2014 when extensive research into and application of related technologies (e.g., fMRI and working memory) first occurred. In particular, fMRI has been prevalent and has guided the development of research experiments in various directions on a large scale. For example, researchers have explored the problems of habituating security warnings (Vance et al., 2018), the potential to induce trust between individuals and their avatars (Riedl et al., 2014), and the potential to induce trust between individuals and their avatars (Xu et al., 2020). Behavior, decision support, and information systems have become popular research topics in numerous emerging research fields.

Eye-tracking was introduced in 2017, greatly expanding the related technologies and research methods. Eye-tracking allows for experimental designs that focus on more specific and subtle dimensions in neuroscience, such as cognitive fit and memory (Walden et al., 2018; Bera and Poels, 2019) (including but not limited to working memory). Under these basic conditions, the research direction in this field has been continually extended. Many research objectives and experimental designs have been based on a comprehensive analysis of customer psychology. The promotion of neuroscience technology has also facilitated the analysis and comprehension of user psychology.

## Discussion and Conclusion

### Research Discussion

Neuroscience attracted great attention from scholars in the field of information systems (IS) a decade ago when Dimoka et al. (2009) systematically introduced the concept of NeuroIS. According to the definition by Riedl et al. (2010), NeuroIS is the application of neuroscience and neurophysiological theories and tools in order to “facilitate scientific progress in the IS discipline.” Thus, the present study analyzed 99 articles on NeuroIS retrieved from the WOS database between 2010 and 2021 to provide a deeper insight into the research field. The conclusions drawn from the analysis results are as follows:

The co-author network helps to identify leading researchers and institutions. Angelika Dimoka and René Riedl are two prominent scholars based on their total citations. Both proposed a profound research agenda and outlook for NeuroIS in the early 2010s.

The number of articles published each year has increased steadily, indicating that NeuroIS has experienced preliminary exploration of research hotspots at present. Before 2014, most studies were limited to cognitive neuroscience, and the main research topics focused on information search and price premiums. In 2014, fMRI as well as other related techniques and approaches were extensively used to design and conduct experiments. Later, eye-tracking greatly expanded the methodology of NeuroIS in 2017, which focuses more on the specific and micro dimensions (e.g., cognitive fit and memory). Accordingly, numerous studies have started to apply these techniques to further analyze and comprehend consumer behavior and information processing. For instance, Hong et al. (2021) contributed to this strand of literature by identifying the role of animated banner ads in online marketing with eye-tracking. The advantages and disadvantages of these tools have also been discussed (Dimoka et al., 2012, respectively).

From the perspective of keywords, the most important three clusters are “cognition and behavior,” “research methodologies and tools,” and “research agenda and potential directions.” In addition, many studies have examined the topics of task (e.g., Kim et al., 2015; Cheung et al., 2017; Ahn et al., 2018; Browne and Walden, 2020), trust (Dimoka, 2010; de Guinea et al., 2014; Karampournioti and Wiedmann, 2021), validity (Twyman et al., 2014a,b; Moody and Galletta, 2015), and working memory (Li et al., 2014; Berget and Sandnes, 2016; Jenkins et al., 2016), which may still be hot topics for future research.

According to the cited reference (co-citation), the articles obtained can be divided into three categories, from macro to micro, roughly consistent with the results of word clustering. Some articles cited reviews on NeuroIS or IS and discussed the research field at an aggregate level. Another type of article mainly contributes to NeuroIS by introducing and improving methodologies and techniques. These tools, such as fMRI, EEG, EFRP, and Brownie, as a platform for conducting NeuroIS experiments (Hariharan et al., 2017), to some degree, imply the research landscape of NeuroIS studies. To further understand the hot topic of the time change, keyword emergence analysis was conducted on the progress of keywords in this study. Based on the analysis results, it can be concluded that, from 2010 to 2013, there were few research fields on neural information systems, and researchers focused on the discussion of cognitive neuroscience-related issues during this period. Later, because several special journals in the field of Information Systems were published in 2014, a large number of studies began to apply fMRI and other research tools. In 2017, NeuroIS embraced the application of technology and eye-tracking, and the research direction was continuously extended. Many experimental designs have been based on a complete analysis of consumer psychology. Compared with the initial research introduced from neuroscience to information systems, the research on NeuroIS was more focused on neurocognitive science, and subsequent research on NeuroIS was further explored by information system researchers. Through content analysis of 99 papers, this study also made several important findings. For example, in the selection and adoption of research tools, eye-tracking is currently the main core tool in the field of information systems, especially for online marketing and web search tasks, followed by tools such as fMRI and EEG/ERPs. The research results in selecting research methodology also show that mixed research was mainly applied after 2020, which also shows that Information Systems researchers attach more importance to the mixture of multiple methods in behavior measurement.

### Implications for Academic Research

Research on neural information systems is mainly related to information search, human-computer interaction, e-commerce, and other issues. Such issues are related to consumer decision-making and behavior. Undoubtedly, with the development of the information systems field, this topic has become more important. Bibliometric and content analyses were used to summarize the application of neuroscience in IS research on Information Systems. The following important research prospects and directions are proposed in this study to provide more detailed research directions for researchers in the NeuroIS field.

First, from the perspective of the development of research trends, under the prospect of more and more interdisciplinary integration perspective, apart from the traditional investigation and experiment methods, the research on online purchase has become a new research channel to explore technology adoption, purchase behavior and consumer intention through the tools of neuroscience in major NeuroIS studies. It can also improve consumers’ purchase intention to determine the influence of purchase behavior by studying buyers’ experiences or decision-making (Xu et al., 2020; Karampournioti and Wiedmann, 2021).

Second, topics such as network security and fake news are also important research directions in the NeuroIS field, particularly in the discussion of social media user behavior (Moravec et al., 2019), information security (Twyman et al., 2014b; Anderson et al., 2016b; Warkentin et al., 2016) and phishing (Jaeger and Eckhardt, 2021). Based on the above two research directions, this study suggests that NeuroIS will remain focused on online buyer decision-making behaviors in the future, especially in the directions of Livestream e-commerce and short video streaming, through which changes in consumer behavior and purchase decision making could be further explored. With regard to information security, discussions of fake information on social media and fake advertisements on social media will continue.

Finally, future research should integrate multiple neuroscientific tools and methods. It can be found that the use of neuroscience tools is gradually diversified. In particular, mixed research methods will gradually become popular in 2020 (Ellison et al., 2020; Zhang and Zhang, 2021). Whether it is empirical research combined with neuroscience experiments, traditional experiments combined with neuroscience experiments, or the integration of multiple neuroscientific experimental tools, it can be found that mixed research is relatively new in NeuroIS. In part, this shows that mixed research is a new trend in NeuroIS. The prediction of consumer behavior can more accurately analyze the impact of consumer behavior decisions.

### Research Limitation

However, research limitations cannot be avoided. Although this study used ABS 3-star journals in the field of information systems as the main data source, there are still some limitations. In particular, NeuroIS journals are not limited to 3-star journals. The Journal of Electronic Commerce, Electronic Commerce Research, Frontiers in Neuroscience, Journal of Advertising Research, European Journal of Marketing, Journal of Consumer Psychology, Journal of Interactive Marketing, Journal of Consumer Research, and other journals in the field of e-commerce or marketing have also studied NeuroIS. In addition, the Notes in Information Systems and Organization collected at the NeuroIS Retreat conferences and NeuroIS Society also has a number of NeuroIS-related issues. These studies were not included in this study and can be further analyzed and discussed in future studies.

## Data Availability Statement

The original contributions presented in this study are included in the article/supplementary material, further inquiries can be directed to the corresponding author/s.

## Author Contributions

C-LL and ZC designed the research and provided guidance throughout the entire research process. C-LL, ZC, and XJ collected the references, did the literature analysis, and wrote the manuscript. GC helped translating and offered modification suggestions. XJ participated in the collecting, analyzing, and organizing of the literature. All authors contributed to the article and approved the submitted version.

## Conflict of Interest

The authors declare that the research was conducted in the absence of any commercial or financial relationships that could be construed as a potential conflict of interest.

## Publisher’s Note

All claims expressed in this article are solely those of the authors and do not necessarily represent those of their affiliated organizations, or those of the publisher, the editors and the reviewers. Any product that may be evaluated in this article, or claim that may be made by its manufacturer, is not guaranteed or endorsed by the publisher.
